# Polypharmacy and Drug–Drug Interactions in Chronic Obstructive Pulmonary Disease: A Narrative Clinical Review

**DOI:** 10.3390/pharmaceutics18060640

**Published:** 2026-05-23

**Authors:** Maria-Medana Drăgoi, Florina-Diana Goldiș, Sabina-Oana Vasii, Daiana Colibășanu, Liana Suciu, Angela Caunii, Lucreția Udrescu

**Affiliations:** 1 Center for Drug Data Analysis, Cheminformatics, and the Internet of Medical Things, Victor Babeş University of Medicine and Pharmacy Timişoara, 300041 Timişoara, Romania; medana.tuica@umft.ro (M.-M.D.); diana.goldis@umft.ro (F.-D.G.); sabina.vasii@umft.ro (S.-O.V.); daiana.handa@umft.ro (D.C.); udrescu.lucretia@umft.ro (L.U.); 2 Doctoral School of Pharmacy, Victor Babeş University of Medicine and Pharmacy Timişoara, 300041 Timişoara, Romania; 3 Department II—Pharmacology, Physiology and Physiopathology, Victor Babeş University of Medicine and Pharmacy Timişoara, 300041 Timişoara, Romania; 4 Department I—Clinical Pharmacy and Drug Analysis, Victor Babeş University of Medicine and Pharmacy Timişoara, 300041 Timişoara, Romania

**Keywords:** drug safety, polypharmacy, regimen complexity, pharmacokinetic interactions, inhaled therapy, older adults, palliative care, exacerbations

## Abstract

**Background**: Chronic obstructive pulmonary disease (COPD) is commonly managed alongside multimorbidity, polypharmacy, recurrent treatment escalation, and older age, all of which increase vulnerability to drug–drug interactions (DDIs). We aimed to synthesize the main DDI domains relevant to COPD pharmacotherapy and to distinguish harmful DDIs from beneficial combination therapy and formal compatibility findings. **Methods**: We performed a narrative review using structured literature searches and citation tracking to evaluate COPD-related studies. We prioritized direct COPD-specific DDI evidence, while also including mechanistic, class-specific, and contextual studies when direct evidence was lacking. Retained evidence included observational cohorts, prescribing studies, pharmacokinetic trials, case reports, and systematic reviews. **Results**: The reviewed literature indicates that DDI vulnerability in COPD is driven less by isolated drug pairs than by overall regimen complexity, multimorbidity, aging, fragmented prescribing, and high-intensity treatment periods such as exacerbations, hospitalization, and discharge. Key DDI domains included cardiopulmonary co-treatment, QT-related vulnerability, and potential or clinically relevant interactions amplified during exacerbations. Inhaled therapies are not universally interaction-free, particularly with strong metabolic inhibitors. Psychotropics, frailty, dementia, and palliative care further increase clinical complexity. However, beneficial bronchodilator combinations and formal compatibility studies demonstrate that not all multidrug COPD regimens are harmful. **Conclusions**: In COPD, DDI assessment should focus on the full treatment regimen and not be limited to a set of iconic drug pairs. Clinicians must focus on exacerbation-related prescribing, QT-active drugs, theophylline exposure, psychotropic co-medication, and vulnerable subgroups such as older, frail, and palliative patients. Pharmacist-supported drug review, drug reconciliation, and selective deprescribing are key strategies for reducing clinically relevant DDI burden in COPD.

## 1. Introduction

Chronic obstructive pulmonary disease (COPD), a major contributor to health issues worldwide, which leads to high levels of illness and death, is characterized by a heavy symptom burden, frequent exacerbations, and considerable demands on healthcare resources [1,2,3]. COPD pharmacotherapy is based on stratified GOLD standards, employing long-acting bronchodilators as maintenance medication, inhaled corticosteroid combinations for specific patients, and additional drugs for exacerbation and symptom relief based on phenotype and comorbidities [2,4,5]. As a result, COPD pharmacotherapy rarely occurs in isolation from broader multimorbidity management. This complexity is clinically important because many patients with COPD also have other chronic conditions, particularly cardiovascular, metabolic, skeletal, and neuropsychiatric disorders [6,7,8,9,10]. COPD is increasingly recognized as a part of a multimorbid clinical state influenced by shared risk factors and overlapping therapeutic needs [6,10,11,12,13]. In COPD patients, polypharmacy is common and often substantial, especially in the elderly, and co-prescription of drugs with overlapping adverse effects is well documented [12,14,15,16,17]. Consequently, DDI risk in COPD should be interpreted at the regimen level, integrating pharmacokinetic mechanisms, pharmacodynamic overlap, and comorbidity-driven prescribing [15,18,19,20,21,22].

The DDI relevance is amplified at points of therapeutic escalation. During acute exacerbations, the addition of antibacterial therapy may introduce a two-way risk of DDI with drugs used for common comorbidities, creating the potential for either toxicity or therapeutic failure [5,18,23]. In advanced disease, palliative symptom management can further increase interaction liability by layering symptom-directed medicines onto already complex COPD regimens [24,25,26]. At the same time, drug combinations are central to COPD care, and not all multidrug regimens imply harmful interaction liability; clinically useful evidence therefore needs to distinguish true interaction signals from explicit compatibility findings, particularly for inhaled combinations that are routinely used in practice [4,27].

The literature on DDIs in COPD is heterogeneous in both design and reporting style. Relevant signals are scattered across pharmacokinetic studies, case reports, prescribing analyses, observational burden studies, and targeted reviews, while the level of detail reported in abstracts varies substantially [14,18]. Moreover, DDI risk in COPD is not reducible to a short list of iconic drug pairs. Some DDIs are pharmacokinetic, involving altered absorption, metabolism, or clearance; others are pharmacodynamic, arising from overlapping effects on heart rate, potassium balance, QT interval, bleeding, sedation, or anticholinergic burden. Furthermore, some apparent interaction problems reflect the broad multimorbid prescribing. Acute exacerbations, hospital admissions, and palliative escalation further increase this complexity by layering antibacterials, corticosteroids, opioids, anxiolytics, or other symptom therapies onto already dense regimens. However, not all multidrug COPD therapies are unsafe, as some combinations are intended for therapy, and some formal compatibility studies have shown no clinically meaningful interaction. Hence, a clinically useful review must distinguish harmful DDI signals from regimen-level complexity and from compatible or beneficial coadministration.

Our narrative review synthesizes the current evidence on DDIs in COPD pharmacotherapy with a focus on three questions: why patients with COPD are inherently vulnerable to drug-related harm, which types of interactions are most frequently reported in the literature, and how clinicians can recognize high-risk drug combinations without overinterpreting or misinterpreting signals from drug interaction checker tools or relying solely on theoretical data. We paid particular attention to cardiopulmonary comorbidity, QT-related vulnerability, exacerbation treatment, psychotropic co-prescribing, legacy xanthine therapy, myths around inhaled drug interactions, and the increased susceptibility of older, frail, and hospitalized patients.

## 2. Scope and Approach of This Narrative Review

We designed this review as a narrative synthesis informed by structured literature searches. The primary search was conducted in PubMed using combinations of Title/Abstract terms related to drug–drug interactions (e.g., drug interactions, drug–drug interactions, potential drug–drug interactions, pharmacokinetic, pharmacodynamic, cytochrome P450, CYP, transporter, physiologically based pharmacokinetic, PBPK) and COPD-specific terms (e.g., COPD, chronic obstructive pulmonary disease, COPD GOLD), combined using Boolean operators (AND/OR). We restricted the search to publications from 1 January 2000 to 24 February 2026. To ensure broader coverage, we supplemented the PubMed search with additional searches in Google Scholar, together with backward and forward citation tracking and manual evaluation of relevant COPD-related studies. This approach allowed inclusion of heterogeneous evidence, including observational studies, pharmacokinetic data, clinical reports, and review articles, reflecting the complexity of DDI assessment in COPD. We included adult COPD-related studies addressing DDIs, potential DDIs, polypharmacy, regimen complexity, COPD exacerbation treatment, comorbidity-related pharmacotherapy, inhaled therapy, oral COPD drugs, psychotropic co-prescribing, cardiopulmonary co-treatment, and vulnerable COPD populations such as older, frail, hospitalized, nursing-home, and palliative-care patients. We excluded studies focused exclusively on asthma or other non-COPD respiratory diseases unless COPD-relevant data were extractable. We also excluded pediatric-only studies, preclinical-only studies without direct clinical interpretability, single-drug efficacy studies without DDI or regimen-safety relevance, and papers lacking sufficient methodological or clinical details. English-language full-text articles were prioritized. Non-English PubMed records were not excluded during title and abstract screening if an English abstract was available and relevant, but were retained only when reliable extraction and interpretation of COPD or DDI-relevant information was possible.

Evidence was evaluated based on clinical relevance and evidentiary strength. Direct studies specific to COPD; large observational cohorts; and research reporting objective exposure changes, monitoring outcomes, treatment failures, or documented toxicity were prioritized as more clinically informative than isolated case reports, theoretical interaction signals, or database-only potential drug–drug interaction (pDDI) outputs. Pharmacokinetic studies primarily informed mechanistic interpretation, particularly when measured exposure changes were reported. Case reports were regarded as signal-generating unless substantiated by biological plausibility, objective clinical findings, dechallenge or rechallenge data, or consistency with broader evidence. Potential drug–drug interactions identified by electronic screening tools or prescribing criteria were classified as potential, not clinically confirmed interactions unless associated with documented harm or clinically actionable monitoring outcomes.

A total of 136 records were identified through structured PubMed searches, supplementary searches, citation tracking, and manual review. After title and abstract screening, full-text assessment, and duplicate removal, 65 core publications were included in the narrative synthesis.

Because COPD symptoms and DDI manifestations may overlap, we interpreted DDI safety signals conservatively. The occurrence of symptoms during co-medication was not considered sufficient evidence of a clinically confirmed DDI unless supported by temporal association with drug initiation, withdrawal, or dose adjustment; biological plausibility; a recognized pharmacokinetic or pharmacodynamic mechanism; documented exposure changes; objective monitoring findings; dechallenge or rechallenge data; or consistency across multiple studies [28,29]. Objective findings, such as QTc prolongation, hypokalemia, altered international normalized ratio (INR), elevated theophylline concentrations, adrenal suppression, pharmacokinetic exposure changes, or documented treatment failure, were considered stronger evidence than nonspecific symptoms alone [29,30,31]. Conversely, symptoms or outcomes that could plausibly reflect COPD severity, exacerbation, aging, frailty, or comorbidities were classified as potential or hypothesis-generating DDI signals, not as clinically confirmed DDIs [28].

For terminology, we use drug–drug interaction (DDI) as an umbrella term for pharmacokinetic or pharmacodynamic interactions that may alter drug exposure, effect, toxicity, or therapeutic performance. A potential DDI (pDDI) refers to a drug combination identified by databases, prescribing criteria, product information, mechanistic reasoning, or co-prescribing patterns without necessarily demonstrating clinical harm. A clinically documented DDI is an interaction supported by objective evidence such as measured exposure change, altered monitoring parameters, dechallenge or rechallenge information, treatment failure, or documented toxicity. Interaction burden describes the cumulative pattern of pDDIs within a regimen. Clinically meaningful vulnerability refers to patient, disease, or regimen-level factors that make a potential DDI more likely to become clinically actionable; these factors include older age, acute exacerbation, baseline QTc prolongation, electrolyte disturbance, renal or hepatic dysfunction, cardiovascular disease, polypharmacy, fragmented prescribing, palliative-care status, and exposure to drugs with overlapping pharmacodynamic effects.

Given the diverse range of study designs, settings, and level of evidence in the literature, this review aims to emphasize clinical interpretation and thematic synthesis of the findings, and not a formal quantitative analysis.

**Figure 1:**
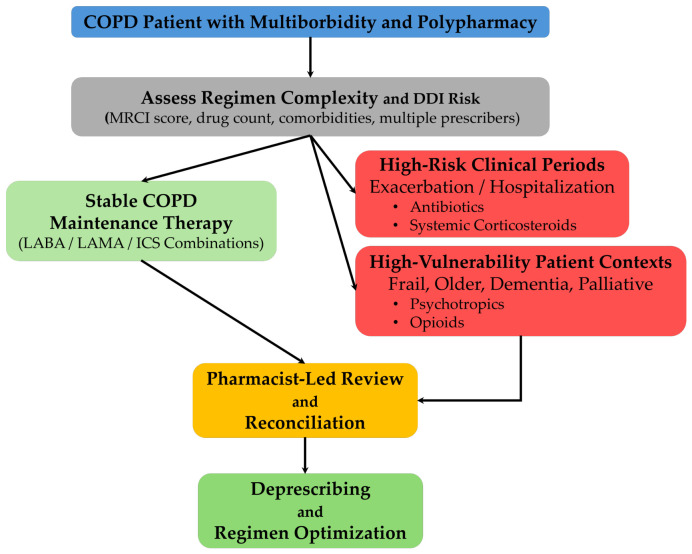
Conceptual framework showing how multimorbidity and polypharmacy in COPD require assessment of regimen complexity and drug–drug interaction risk across stable disease, exacerbation and hospitalization periods, and vulnerable clinical contexts such as frailty, dementia, and palliative care. Pharmacist-supported review, reconciliation, deprescribing, and regimen optimization are key responses to high-risk prescribing moments (COPD, chronic obstructive pulmonary disease; DDI, drug–drug interaction; MRCI, Medication Regimen Complexity Index; LABA, long-acting beta-2 agonist; LAMA, long-acting muscarinic antagonist; ICS, inhaled corticosteroid).

Figure 1 summarizes the regimen-level framework used to organize this review. It highlights how DDI risk in COPD changes across stable disease, exacerbation, or hospitalization, and vulnerable contexts such as frailty, dementia, and palliative care.

## 3. Why COPD Is Intrinsically Vulnerable to DDIs

COPD is particularly vulnerable to DDIs due to its association with chronic respiratory symptoms, recurrent treatment escalation, multimorbidity, and aging [14,15]. At the same time, COPD patients often require a variety of drugs to manage their symptoms and improve their quality of life. These treatments aim at alleviating dyspnea, preventing exacerbations, and reducing airway inflammation [14]; many patients may also bear comorbidities such as cardiovascular diseases (CVDs), diabetes, and osteoporosis, which require their own drug regimens; mental health problems, such as anxiety and depression; and complications like gastroesophageal reflux and chronic pain are also addressed through medication [32,33]. Therefore, the management of COPD patients involves a comprehensive and layered treatment strategy, relying on a complex array of drugs and the use of specialized medical devices. As their condition evolves, the intensity and focus of their treatment may change over time, requiring ongoing adjustments to their management plan to support their health outcomes best. The literature on COPD polypharmacy is highly heterogeneous, yet consistently points in the same direction: patients with COPD are frequently exposed to multiple concurrent drugs, leading to inadequate adherence, adverse drug reactions, and DDI risk [14,34,35,36,37]. In other words, COPD significantly increases the likelihood of DDIs primarily due to the many drugs used, alongside their use in a clinically unstable and multimorbid population.

Primary-care and cohort data support this interpretation. In the Crete study, most patients with COPD had multimorbidity, more than half had polypharmacy, and older adults were affected disproportionately. Ierodiakonou et al. reported that polypharmacy was associated with worse symptom burden, several cardiometabolic disorders, cancer, anxiety, depression, and prostate disease. In older adults, potentially inappropriate drugs were observed in nearly one in ten patients. Coadministration patterns revealed cumulative risks for falls, constipation, and cardiovascular events, and 15 potential DDI pairs in 11.5% of the cohort [32]. These findings show that the risk of DDIs is not limited to tertiary care or acute admissions; even in community-managed COPD, clinically relevant DDI burden is embedded in routine care. This vulnerability also appears to extend beyond hospital settings, as evidence from community pharmacies suggests that clinically relevant interaction burden can be identified in routine outpatient COPD pharmacotherapy [38,39].

Large-cohort evidence adds an important nuance: a heavy medication burden does not automatically translate into extreme rates of severe DDIs, but it does create a background of vulnerability. In the COSYCONET study, the median number of drugs was at least five in all patient categories, with at least three non-respiratory drugs across groups; in older patients, 10.2% were prescribed drugs listed as age-inappropriate in the PRISCUS criteria, 4.2% experienced serious adverse drug combinations, 6.4% had potentially clinically relevant unwanted combinations, and 8.0% had combinations considered wanted [40]. This pattern highlights the complexity of COPD pharmacotherapy, involving a combination of drugs indispensable for the treatment of comorbidities, intentional combinations, age-related prescribing concerns, and a smaller but clinically meaningful subset of potentially harmful combinations.

The concept of drug regimen complexity, instead of drug count alone, is specifically useful in COPD. Negewo et al. demonstrated that patients with COPD had a median of five comorbidities and a median medication regimen complexity index (MRCI) score of 24, and that COPD-specific regimens were more complex than non-COPD regimens despite involving fewer drugs. A higher level of COPD treatment complexity correlated with worse health-related quality of life, more prior exacerbations, lower lung function, and shorter 6-min walk distance; cardiovascular, gastrointestinal, and metabolic comorbidities also contributed materially to higher total regimen complexity [34]. These findings highlight that DDI vulnerability in COPD is related not only to drug count but also to regimen structure: multiple inhaler devices, different dosage forms, frequent add-on therapies, and various non-respiratory drugs create a pharmacotherapeutic environment prone to nonadherence, adverse drug events, and clinically relevant interaction signals.

However, not all patients with COPD are equally vulnerable. Risk appears to cluster in those with more comorbidities, more drugs, and more fragmented prescribing. Stojadinović et al. reported that the most serious potential DDIs were common depending on the drug interaction checking tool used; they also showed that risk increased with increasing drug count, anticoagulant or antiarrhythmic exposure, more comorbidities, and a higher number of independent prescribers—each additional drug roughly doubled the risk of a serious potential DDI [41]. As a result, COPD is intrinsically vulnerable to DDIs because it combines complex standard pharmacotherapy, various comorbidity treatments, exacerbation treatment, and the cumulative effects of aging and fragmented prescribing practices.

Table 1 summarizes key cohort, prescribing, and hospital-based studies to provide quantitative context and to avoid overinterpreting potential DDI signals as confirmed clinical harm. This table distinguishes potential DDIs and co-prescribing risk signals from drug-related problems and clinically documented outcome data.

## 4. Beneficial Interaction, Compatibility, and Harmful DDIs: Defining the Boundaries

A narrative review of DDIs in COPD should distinguish between beneficial therapeutic interactions, formal compatibility, and harmful drug–drug interactions. A beneficial therapeutic interaction refers to intentional pharmacological or clinical complementarity between drugs, such as LABA/LAMA co-administration to improve bronchodilation, symptoms, or exacerbation control. Compatibility means evidence that coadministration does not cause a clinically relevant pharmacokinetic or safety concern under studied conditions, like formal studies of fixed-dose inhaled combinations. Absence of a clinically meaningful DDI means available evidence shows no relevant exposure change, toxicity signal, or loss of efficacy. However, this should not be seen as proof of universal safety in all patients or clinical contexts. In contrast, a harmful DDI increases toxicity, reduces therapeutic efficacy, or creates a clinically actionable risk in a COPD-relevant context. In COPD pharmacotherapy, these categories may overlap because combination treatment is a core component of care. Long-acting bronchodilators with different mechanisms are intentionally combined to improve bronchodilation, symptoms, and exacerbation control, and escalation to triple therapy is often guideline-concordant and should not be interpreted automatically as inappropriate prescribing [45,46,47].

The literature on beneficial interactions supports this distinction. Co-administration of long-acting beta-2 agonist (LABA) and long-acting muscarinic antagonist (LAMA) may produce additive or synergistic bronchodilation [48]. In human isolated bronchi, aclidinium plus formoterol showed synergistic relaxation at low concentrations, consistent with true pharmacologic complementarity [49]. By contrast, umeclidinium plus vilanterol did not show synergism at the marketed delivered-dose ratio, although synergy emerged at low isoeffective concentrations [50]. These findings indicate that combination therapy and harmful DDIs are not opposite ends of a single spectrum.

Formal compatibility studies reinforce this point. In a phase I study, adding budesonide to glycopyrronium/formoterol did not produce a pharmacokinetic interaction, supporting the internal compatibility of fixed-dose triple therapy [51]. Likewise, co-administration of umeclidinium or umeclidinium/vilanterol with verapamil caused only a modest increase in umeclidinium exposure and no clinically significant increase in vilanterol exposure [52]. Although such studies are limited by healthy-volunteer designs and PK-focused endpoints, they help avoid an overly hazard-centered interpretation of all multidrug COPD regimens.

At the same time, therapeutic benefit does not eliminate safety considerations. Meta-analytic evidence suggests that some long-acting bronchodilator combinations may have different cardiovascular risk profiles depending on the comparator and baseline patient risk [53], while pharmacovigilance data indicate that different single-inhaler triple therapies may show different patterns in real-world adverse events, including pneumonia, candidiasis, device-related problems, and vascular signals [54]. These findings do not negate the clinical value of multidrug inhaled therapy, but they underscore that compatibility and benefit should not be equated with absence of harm.

This distinction is essential to avoid the misconception that all multidrug COPD therapy is intrinsically unsafe.

## 5. Major DDI Domains in COPD Pharmacotherapy

Before considering individual clinical domains, it is useful to separate the main mechanisms through which DDIs become relevant in COPD. Pharmacokinetic mechanisms include altered drug exposure through metabolic inhibition or induction, especially CYP-mediated pathways such as CYP1A2, CYP2D6, and CYP3A4. They also include transporter-mediated effects involving systems like P-glycoprotein, OATP, or BCRP for selected co-medications [55]. These mechanisms are most relevant to exposure-sensitive drugs such as theophylline, roflumilast, inhaled corticosteroids in the presence of strong CYP3A4 inhibitors, selected psychotropics, anticoagulants, statins, and anti-infectives [22,27]. Pharmacodynamic mechanisms are often more clinically visible in COPD. They include cumulative QT liability, potassium loss, bleeding risk, sedation or respiratory depression, anticholinergic burden, duplicated bronchodilator effects, and overlapping cardiovascular effects [18,24,56]. In COPD, these mechanisms rarely occur in isolation; they interact with exacerbation status, hypoxemia, cardiovascular comorbidity, renal or hepatic dysfunction, frailty, and fragmented prescribing.

Table 2 summarizes the main pharmacokinetic and pharmacodynamic mechanisms relevant to DDI assessment in COPD. The table provides a concise framework linking drug classes, interaction pathways, clinical consequences, and COPD-relevant contexts.

### 5.1. Cardiovascular Comorbidity and Cardiopulmonary Interaction Burden

In COPD, the interrelation between respiratory and cardiovascular pharmacotherapy is one of the most clinically important. CVDs are highly prevalent in COPD, which profoundly shapes prescribing. Corrao et al. note that approximately 64% of patients with COPD are treated for concomitant CVDs and that about one-third die from cardiovascular causes [57]. This conclusion turns COPD pharmacotherapy into a cardiopulmonary balancing problem rather than a single-organ prescribing exercise.

The clinical tension is most evident with beta-blockers. Historically, concern about bronchoconstriction led to beta-blocker underuse in COPD, even in patients with clear cardiac indications. However, both Corrao et al. and Lipworth et al. argue that this reluctance to use beta1-selective blockers is often not justified. Corrao et al. summarize evidence that cardioselective beta blockers do not significantly attenuate the bronchodilator response to inhaled beta-2 agonists and may even be associated with reduced mortality and fewer respiratory admissions [57]. Lipworth et al. similarly stress that beta-blockers remain underused despite guideline support and that beta1-selective drugs such as bisoprolol, nebivolol, and metoprolol are preferred over nonselective agents because they are less likely to provoke bronchoconstriction [58].

The best interpretation of this evidence is therefore not that beta blockers and bronchodilators are mutually incompatible, but that agent selection and context matter. Olschewski et al. further argue that this cardiopulmonary treatment is pharmacologically rational: in COPD patients with heart failure or ischemic heart disease, beta1-selective beta blockers should still be used when necessary, while inhaled LABAs and LAMAs can also be prescribed in symptomatic disease [59]. They also note that other cardiac drugs create pulmonary problems of their own: anticoagulants may increase bleeding complications in susceptible patients, and amiodarone can cause dose-dependent pulmonary toxicity that may mimic or worsen respiratory decline.

Hospital-based pDDI data show how these pharmacologic tensions appear in real prescribing patterns. In a study of hospitalized patients with chronic heart failure and COPD, Roblek et al. found a median of 6 drugs on admission and 7 at discharge, with 6.5 to 7.2 potential DDIs per patient. Type X interactions (i.e., the highest risk drug combinations that should be avoided) were rare overall. However, the most common type X interaction was when combining a nonselective beta blocker with a beta-2 agonist. More frequent type C combinations (i.e., clinically significant interactions that require monitored therapy) included loop diuretic plus beta-2 agonist, beta-2 agonist plus beta-2 agonist, beta-2 agonist plus theophylline, and diuretic plus corticosteroid—all patterns reflecting potential pharmacodynamic vulnerability during cardiopulmonary co-treatment [19]. Patients with both heart failure and COPD had more clinically relevant potential DDIs than patients with either condition alone.

Altogether, the cardiopulmonary literature suggests that cardiovascular comorbidity is one of the main engines of DDI complexity in COPD. Therefore, the practical conclusion is that COPD patients with CVDs require integrated cardiopulmonary prescribing, not therapeutic retreat from either side.

### 5.2. QT Prolongation as a Recurring COPD Risk Phenotype

QT prolongation is an important COPD risk domain because it links respiratory pharmacotherapy, psychotropic co-prescribing, anti-infective exposure, electrolyte disturbance, and cardiovascular comorbidity. Several drug classes discussed in the COPD DDI literature, including beta-2 agonists, macrolides, fluoroquinolones, antidepressants, antipsychotics, and selected antifungals, may prolong ventricular repolarization or amplify arrhythmic risk through effects on autonomic tone, electrolytes, or drug metabolism.

Population data support the argument that QT vulnerability is intrinsic to COPD and not simply a byproduct of overt cardiac disease or hospital-level prescribing. In a Swedish study by Nilsson et al., the prevalence of borderline and prolonged QTc increased with the GOLD stage, and in participants with COPD, prolonged QTc was associated with worse survival over follow-up [60]. This prognostic association was not observed in normal lung function or restrictive spirometric pattern comparison groups, suggesting that QT prolongation has a distinct clinical meaning in COPD itself and does not function as a nonspecific marker of cardiometabolic burden.

QT prolongation in COPD should not be interpreted uniformly as evidence of a confirmed DDI or as a disease-specific signal. In individual patients, particularly when QTc prolongation is borderline or isolated, it may reflect an incidental ECG finding, a measurement-related artifact, or serve as a marker of acute illness, hypoxemia, cardiovascular disease, electrolyte imbalance, or increased COPD severity [44,60]. In this review, we use the term QT-related risk phenotype to describe a recurrent clinical pattern, not merely a single ECG abnormality [44,60]. The clinical significance of the signal increases when prolonged QTc is observed in conjunction with exposure to QT-active or electrolyte-altering medications, such as beta-2 agonists, macrolides, fluoroquinolones, antipsychotics, antidepressants, diuretics, systemic corticosteroids, antifungals, or antiarrhythmic agents; during periods of exacerbation or hospitalization; or in patients with pre-existing cardiac vulnerability [20,24,44,61,62]. This distinction prevents the over-attribution of all QTc abnormalities to DDIs while acknowledging that individuals with COPD may accumulate multiple QT-relevant risks within a single therapeutic regimen.

Acute exacerbation studies extend this observation into the hospital setting. For example, in a prospective cohort study by Zilberman-Itskovich et al., 35.8% of inpatients with acute exacerbation of COPD had prolonged QTc on admission, and all in-hospital deaths occurred in the prolonged-QTc group [44]. Importantly, the study did not find meaningful differences in potassium, magnesium, calcium, renal function, or blood gases between patients with prolonged and normal QTc, and the authors concluded that QT prolongation in this setting was not explained by electrolyte disorders alone.

Another respiratory-inpatient study by Spanakis et al. provides a complementary drug-centered perspective. In their cohort, 61% of patients had polypharmacy upon admission, 98% during hospitalization, and 63% at discharge, whereas potential DDIs were in 55%, 96%, and 63% of patients at those same time points. Most DDIs were pharmacodynamic, and potential QT prolongation was the most common mechanism [20]. The authors also note that clinically significant DDIs were observed particularly at admission and discharge, associated with outpatient prescribing and transition-of-care periods as high-risk moments.

These studies indicate QT prolongation as a COPD-relevant risk marker whose clinical interpretation depends on the drug regimen, exacerbation status, and baseline cardiovascular vulnerability.

### 5.3. Exacerbations, Hospitalizations, and DDI Amplification Associated with Anti-Infectives

Acute exacerbations and hospital admissions are among the most important factors that can amplify DDIs in COPD patients. These episodes require intensifying bronchodilator treatment and adding systemic corticosteroids, antibacterials, oxygen, and various supportive therapies; this pharmacotherapeutic approach is challenging in patients who already receive multiple chronic treatments for cardiovascular, metabolic, psychiatric, or gastrointestinal comorbidities. Therefore, the issue of DDIs during exacerbation care is different from that in stable COPD: new short-term drugs are introduced rapidly, prescriber teams multiply, and the regimen changes repeatedly during admission and discharge [42].

The systematic review by Wang et al. directly addresses this issue by showing that antibacterials prescribed in COPD exacerbations may generate potential or clinically relevant DDIs with a wide range of drugs used for comorbidities, and that these interactions can lead both to toxicity and to therapeutic failure. The review structured the literature into two broad patterns—co-medications altering the pharmacokinetics of antibacterials, and antibacterials altering the pharmacokinetics of co-medications. The study highlighted that COPD exacerbation prescribing cannot be approached as if antibiotics operate in isolation from the rest of the regimen [18].

Hospital-based studies reinforce the idea that exacerbations represent high-risk situations for medication management. In a Chinese study reported by Li et al., 393 patients admitted with acute exacerbation of COPD generated 640 drug-related problems, most commonly in the domain of treatment safety. The drug classes frequently implicated were antibiotics, corticosteroids, and proton pump inhibitors, and the risk increased in patients hospitalized over eight days, on ten or more drugs, or with renal dysfunction [42]. These data shift the discussion from focusing exclusively on specific DDIs to a broader understanding of prescribing practices for acute COPD.

The study by Spanakis et al. on a cohort of hospitalized respiratory patients showed that polypharmacy and DDIs increased during hospitalization, with pharmacodynamic interactions being the most prevalent; QT prolongation, INR modulation, and CYP-mediated inhibition were the most common mechanisms [20]. Although the cohort was not COPD-exclusive, COPD exacerbation was among the common admission diagnoses, and the pattern is highly applicable to hospitalized COPD care. These findings also help explain why discharge is such an important and underemphasized moment in COPD drug safety [20]. Li et al. showed that interventions led by pharmacists received a high level of acceptance and that most drug-related problems were solved, highlighting that structured medication review can significantly reduce harm in hospitalized COPD patients [42]. As such, exacerbations and hospitalization should be understood as high-intensity pharmacotherapeutic windows in COPD, as they are the contexts in which additional required drugs and comorbidity drugs most often converge and where pharmacokinetic and pharmacodynamic DDIs become clinically relevant.

A similar amplification of interaction burden may occur in less common but clinically important pulmonary infection scenarios, such as aspergillosis complicating structurally diseased or steroid-exposed lungs, where azole therapy can add substantial pharmacokinetic and pharmacodynamic interaction risk and may require multidisciplinary review [63]. Acute infectious overlays can further intensify this problem, as highlighted during the COVID-19 era, when temporary treatment regimens created additional interaction concerns in patients with COPD and other comorbidities [64].

### 5.4. Xanthines and PDE4 Inhibitors: From Theophylline Burden to a More Nuanced Roflumilast Profile

Among oral COPD drugs, theophylline remains the clearest example of a legacy therapy with narrow therapeutic index, frequent adverse effects, and clinically important DDI liability [65]. Although it has oral availability and bronchodilator or anti-inflammatory effects, it is less favored than modern inhaled therapies and requires plasma-level monitoring in clinically relevant situations.

The comparison with doxofylline is useful because it shows that xanthine-related interaction burden is not uniform across the class. In two review articles, doxofylline is presented as pharmacologically and pharmacokinetically distinct from theophylline: unlike theophylline, doxofylline does not significantly interfere with CYP1A2, CYP2E1, or CYP3A4 and therefore appears substantially less prone to interacting with drugs metabolized through those pathways [56,65]. The same reviews also stress that doxofylline has a better tolerability profile, less cardiac stimulation, less gastric toxicity, and no need for routine therapeutic drug monitoring.

The importance of theophylline in a DDI analysis lies not only in the fact that it is an older bronchodilator, but in the fact that it functions as an interaction hub. As an example, Weiss et al. describe theophylline as a narrow therapeutic index drug that causes both pharmacokinetic and pharmacodynamic DDIs [24]. Theophylline is identified in studies reviewing checker-based burden, prescribing in the elderly, and cardiopulmonary literature as a drug whose risk profile exceeds its current therapeutic relevance.

By contrast, the interaction profile of roflumilast—a selective phosphodiesterase-4 (PDE4) inhibitor indicated to treat exacerbations in severe COPD [66,67]—is more nuanced and substantially less alarming than a simple extension of theophylline logic would imply. The clearest clinically relevant signal is enzyme induction. In a study investigating the interaction between rifampicin and roflumilast, the anti-infective co-administration reduced roflumilast AUC by 80%, and total PDE4 inhibitory activity by about 58%, suggesting a risk of diminished therapeutic efficacy [68]. Inhibition studies point in the opposite direction but are less alarming clinically. With enoxacin, roflumilast exposure increased by about 56% and total PDE4 inhibitory activity by about 25%, yet this was interpreted as a weak interaction unlikely to be clinically relevant [69]. Similarly, cimetidine increased roflumilast exposure and raised total PDE4 inhibitory activity by 47–48%, but the study concluded that dose adjustment was not required [70]. These studies emphasize that roflumilast is not interaction-free, and modest inhibitor-mediated exposure increases do not appear to present the same practical fragility as classic theophylline DDIs.

Reassuring compatibility data strengthen this interpretation further. In the midazolam interaction study, therapeutic steady-state concentrations of roflumilast and roflumilast N-oxide did not alter the disposition of the CYP3A substrate midazolam, suggesting low susceptibility to alter the clearance of other CYP3A4-metabolized drugs [71].

The available evidence supports the finding that the interaction profile of roflumilast is more selective and generally less problematic than that of theophylline.

### 5.5. Inhaled Therapies, Corticosteroids, and the Myth of Purely Local Treatment

Inhaled therapy is vital for the management of COPD. It is generally considered safe concerning DDIs due to low systemic exposure and delivery to the target organ. However, this assumption is only partially accurate. Although inhaled delivery reduces first-pass metabolism and limits systemic exposure, it does not eliminate the potential of pharmacodynamic DDIs nor prevent class-related systemic adverse effects. In addition, metabolic inhibition can convert an inhaled drug into a clinically important systemic exposure. The study conducted on community pharmacies by Tezcan and Yaban showed that inhaler drugs are widely used and can potentially lead to DDIs, particularly in COPD patients who have multiple comorbidities and are on several medications [21].

Real-world adverse drug reaction data reinforce this point. In the emergency department analysis by Bergs et al., two-thirds of the included COPD cases were receiving inhaled therapy, and 16% of these inhaled regimens were classified as overdosed. Overdosed cases were more likely to present with malaise and local symptom complexes. Inhaled anticholinergics were reported to cause more local symptoms resembling dysphagia, and inhaled beta-2 agonists caused more sympathomimetic-like and local complaints [72]. These adverse effects were rarely attributed to inhaled therapy by either health professionals or patients.

The inhaled bronchodilator classes illustrate two different mechanisms by which local treatment can still matter systemically. For inhaled beta-2 agonists, the main issue is usually pharmacodynamic overlap. Weiss et al. note that beta agonist toxicities such as tachycardia may be enhanced by tricyclic antidepressants, stimulants, and nabilone, and that concomitant corticosteroids may further aggravate hypokalemia [24]. Inhaled muscarinic antagonists rarely cause clinically relevant CYP-mediated pharmacokinetic interactions, and their bronchodilator effect is predominantly local. However, systemic exposure is not absent and may vary according to molecule, device, dose, and patient factors such as renal impairment [73]. Product labels for inhaled anticholinergics caution against concomitant use with other anticholinergic-containing drugs because additive anticholinergic effects may occur [74,75,76,77]. Accordingly, the main concern is cumulative pharmacodynamic anticholinergic burden. This concern is most relevant in susceptible patients, especially older, frail, cognitively impaired, or renally impaired individuals, and in those receiving systemic anticholinergic or anticholinergic-like drugs such as tricyclic antidepressants, antipsychotics, antiemetics, opioids, bladder antimuscarinics, antiparkinsonian agents, or antisecretory agents.

The most clinically explicit exception to the purely local assumption concerns inhaled corticosteroids in the presence of strong CYP3A4 inhibition. The case report on ritonavir–fluticasone combination is especially instructive, representing a clinically documented pharmacokinetic DDI: in a patient with HIV and COPD, the combination of lopinavir/ritonavir with inhaled fluticasone/salmeterol resulted in severe osteoporosis, iatrogenic Cushing syndrome, and adrenal insufficiency [78]. The report explains that CYP3A4 extensively metabolizes fluticasone and that ritonavir—a potent CYP3A4 inhibitor—can dramatically increase fluticasone exposure, turning a therapy normally regarded as locally delivered into a source of systemic glucocorticoid toxicity.

At the same time, the interaction profile of inhaled therapy should not be overstated. Studies on the compatibility of modern inhaled combinations are often favorable, making the distinction between beneficial combination therapy, PK compatibility, and harmful DDI crucial. The risk of harm rises significantly when inhaled therapy is combined with other drugs for multiple comorbidities, especially in cases of overdose or introduction of strong metabolic inhibitors.

## 6. Psychiatric Comorbidity and Psychotropic-Related Interaction Burden

Psychiatric comorbidity is clinically important in COPD because anxiety and depression influence symptom perception, self-management, exacerbation risk, healthcare use, and medication burden [79]. This issue complicates DDI interpretation, since psychotropic prescribing is often added to an already complex respiratory and cardiometabolic regimen.

In a primary-care study, polypharmacy in COPD was associated not only with cardiometabolic disease and cancer, but also with depression and anxiety; among patients aged 65 years or older, potentially inappropriate medications were found predominantly among mental-health drugs [32].

A mismatch between clinical need and disease-specific evidence has long characterized the literature on the treatment of anxiety and depression in COPD. Cafarella et al. noted that pharmacologic treatment of anxiety and depression in COPD has historically relied on a relatively thin evidence base, with small trials, short follow-up periods, and high dropout rates [80]. Selective serotonin reuptake inhibitors (SSRIs) were generally preferred first-line antidepressants in COPD, but this preference rested more on extrapolation from general psychiatric practice than on robust COPD-specific trials. Benzodiazepines, commonly used for anxiety, lack substantial randomized evidence in COPD and are best indicated for short-term or acute use instead of routine long-term treatment.

More recent reviews emphasized the clinical caution around the use of antidepressants, without advocating for a categorical interdiction. Kaplan argues that antidepressants remain important for many patients with COPD and depression, but observational data raise safety concerns. Large studies in older adults associated the initiation of new SSRIs and serotonin-norepinephrine reuptake inhibitors (SNRIs) with higher rates of hospitalization for COPD or pneumonia, emergency respiratory visits, and respiratory-specific events, as well as all-cause mortality [81]. These findings do not establish causality but suggest caution against considering antidepressants as harmless in older, frail, or vulnerable patients. Furthermore, Siraj reaches a similar balanced conclusion, supporting pulmonary rehabilitation, cognitive behavioral therapy, and supportive counseling as important components of care, while acknowledging that SSRIs and SNRIs are often used in moderate to severe disease or when non-pharmacologic strategies prove inadequate [79]. Bhattacharyya et al. caution against polypharmacy, recommend judicious use of benzodiazepines and antipsychotics at their lowest effective dose for the shortest possible period, and adapt doses to age, weight, and clinical setting [82].

The risk of DDIs associated with psychotropics in COPD extends beyond QT prolongation to include CYP-mediated pharmacokinetic mechanisms, particularly for antipsychotics and antidepressants. Risperidone and aripiprazole are strongly linked to CYP2D6 metabolism, quetiapine mainly to CYP3A4, and clozapine and olanzapine to CYP1A2-mediated pathways [83,84]. Strong CYP2D6 inhibitors, such as fluoxetine, paroxetine, and bupropion, may increase exposure to CYP2D6-metabolized antipsychotics, while CYP3A4 inhibitors, including some azole antifungals and macrolides, may increase exposure to CYP3A4 substrates [85]. Recent literature supports the clinical relevance of CYP2D6 variability for antipsychotic exposure and adverse effects, although associations vary by drug, outcome, and study design [84,86,87]. In COPD, increased psychotropic exposure may amplify sedation, falls, delirium, extrapyramidal effects, QT liability, and altered respiratory symptom perception, especially in older, frail, hospitalized, or exacerbating patients; population-based COPD data also associate antipsychotic use with an acute, dose-dependent increase in acute respiratory failure risk [88].

Recent psychopharmacology research supported multidomain risk assessment frameworks that combine DDIs, QT liability, anticholinergic burden, serotonergic exposure, and polypharmacy into a single clinically interpretable signal, hence underscoring that psychotropic risk in COPD is unlikely to be captured by any single drug pair alone [89].

The literature highlights that anxiety and depression in COPD patients worsen the outcomes independently, and psychotropic treatment often adds to already complex regimens. The practical implication is not that psychotropics should be avoided, but that the treatment of psychiatric comorbidity in COPD should be individualized and closely monitored.

## 7. Special Populations and Care Settings

### 7.1. Older Adults

Older adults represent a distinct high-risk subgroup in any review of DDIs in COPD because aging changes not only the prevalence of comorbidity and polypharmacy, but also the clinical meaning of a given drug exposure. Geriatric pharmacotherapy research has long shown that inappropriate prescribing in older adults includes not only overtly unsuitable drugs, but also drug–drug and drug–disease interactions emerging from polypharmacy and prescribing fragmentation [90]. Matera et al. emphasize that older COPD patients are often underrepresented in clinical trials because of comorbidities and exclusion criteria, limiting the generalizability of pharmacokinetic (PK) and pharmacodynamic (PD) findings from younger populations [15]. Prescribing in older adults with COPD must account simultaneously for altered drug handling, altered receptor response, polypharmacy, comorbidity treatment, inhaler delivery challenges, social context, and adherence. Aging-related pharmacokinetic changes provide an important foundation for this vulnerability. Older adults often experience decreased renal and hepatic clearance, increased body fat, reduced body water, and worsening respiratory mechanics, all of which alter the disposition of inhaled and systemically administered COPD drugs [15]. Older adults are also more exposed to PD interactions involving beta agonists, beta blockers, diuretics, digitalis glycosides, muscarinic antagonists, antidepressants, bladder antimuscarinics, antipsychotics, and CYP3A4-inhibited inhaled corticosteroids [15].

At the same time, not all age-related conclusions are pessimistic: dual bronchodilation appears broadly acceptable across age strata, reinforcing the need to separate age-related vulnerability from the general assumption that multidrug inhaled therapy is intrinsically unsafe.

Newnham’s review of asthma and COPD medications in the elderly is particularly useful because it clearly articulates the mechanism by which common respiratory therapies can become more hazardous with age. His review emphasizes that hypokalemia and electrocardiographic effects induced by beta-2 agonists are particularly relevant in older patients with ischemic heart disease or arrhythmia, and that concomitant diuretics, corticosteroids, and theophyllines may further worsen potassium loss [91]. He also notes that oral and inhaled corticosteroids can potentiate the systemic sequelae of beta-2 agonists and that theophyllines should be prescribed with extreme caution because of their wide adverse-effect profile and marked DDI liability.

Cohort and prescribing-quality data support these age-related concerns. In Romanian primary care, theophylline was still used as bronchodilator monotherapy in some COPD prescriptions, within a broader elderly population in which more than 85% of prescriptions showed medication-related problems [92]. STOPP/START-based assessment in ambulatory older adults from rural Romania also identified theophylline monotherapy for COPD as potentially inappropriate and found omission of regular inhaled bronchodilator therapy in some patients [93]. In COSYCONET, 10.2% of patients aged 65 years or older had at least one potentially problematic PRISCUS-listed medication, and psychiatric drugs represented the largest group among flagged medications occurring in more than five patients [40]. In the Crete study, 83.6% of older COPD patients had multimorbidity, 62.4% had polypharmacy, and potentially inappropriate medication use was mainly related to mental-health drugs [32].

In this context, older adults should be considered not simply as older versions of the standard COPD patient but as a population in whom the entire pharmacologic landscape changes. Reduced clearance and receptor responses, along with more cardiovascular and psychiatric comorbidities, frequent polypharmacy, and broader exposure to potentially inappropriate medication, all mean that modest pharmacokinetic or pharmacodynamic signals may have greater clinical consequences that need to be considered.

### 7.2. Frailty, Nursing Homes, and Dementia

Frailty, living in a home residence, and dementia are also relevant factors for DDI vulnerability in COPD because they combine reduced physiologic reserve, comorbidities, and complex prescribing distributed across multiple specialties and care settings. In this context, the clinical consequences of DDIs may present as delirium, falls, worsening dyspnea, aspiration, functional decline, loss of treatment adherence, or progressive treatment burden. Rowhani and Iglseder described a case report of an 80-year-old woman with advanced COPD, frailty, and extensive multimorbidity. The repeated attempts to rationalize treatment did not achieve a significant drug reduction until the terminal phase, and probable DDIs and adverse effects were observed repeatedly [94]. The authors explicitly frame this not as an isolated incident, but as a recurring challenge in the care of patients with advanced pulmonary disease complicated by frailty and multimorbidity. The nursing home study by Elli et al. extends this problem from a single case to a multicenter older-adult population. In Italian long-term care nursing homes, 306 of 2604 residents had COPD, but only 84 received at least one COPD drug. COPD treatment was markedly less common in residents with dementia than in those without dementia; among those receiving COPD medications, 53.6% had at least one potentially severe DDI involving drugs used for comorbidities. Pharmacodynamic DDIs were the most common, especially those associated with increased risk of QT prolongation, such as beta-2 agonists combined with diuretics, antipsychotics, or antidepressants [43]. These findings are especially valuable because they show that undertreatment and interaction burden can coexist. The evidence from nursing homes also deepens the psychiatric and QT-related themes discussed earlier in the review. In older institutionalized patients, psychotropic use is common. Additionally, beta-2 agonists, diuretics, and antidepressants or antipsychotics can raise concerns even in younger adults. However, in frail residents with dementia, these interactions can be even more significant due to low mobility, hydration, nutritional status, monitoring reliability, and the ability to report symptoms.

### 7.3. Advanced Disease and Palliative Care

Advanced COPD and palliative care add a further layer of interaction complexity as symptom relief, pain management, and quality of life goals often require drugs that are not part of standard maintenance regimens. Weiss et al. show that COPD therapies may interact with opioids, benzodiazepines, antipsychotics, antidepressants, antiemetics, antisecretory agents, and other commonly used palliative medications through both pharmacodynamic and pharmacokinetic mechanisms [24]. In this setting, even modest interaction signals may become more important because of reduced physiologic reserve and because the goal of care may shift from long-term disease modification to immediate symptom control. Palliative care prescribing in COPD, therefore, cannot be interpreted by the same standards as stable outpatient pharmacotherapy. Some DDIs may be beneficial for relieving severe dyspnea, anxiety, or distress, while others may need simplification if they add burden without adequate benefit.

The literature on palliative care advocates a careful yet pragmatic approach to COPD pharmacotherapy, which should prioritize symptoms, drug burden, and the likely time horizon of benefit. Clinicians should remain alert to additive sedation, anticholinergic burden, QT risk, and corticosteroid-related complications.

## 8. Clinical Implications

Our review reveals several important implications.

- DDI assessment in COPD should start with full medication reconciliation, including inhaled therapy, short-term exacerbation treatments, non-respiratory drugs, OTCs, and drugs prescribed by other specialists.
- Clinicians should focus on high-intensity moments of therapeutic change, especially exacerbations, hospital admission, and discharge. Drug reconciliation at admissions and discharge, is more than a procedural formality; it is a central DDI-prevention strategy in COPD care.
- Clinicians should monitor for therapeutic failure and toxicity, since some meaningful DDIs in COPD increase drug exposure, while others reduce efficacy.
- QT-active regimens require careful review in COPD. QT prolongation commonly occurs with bronchodilators, anti-infectives, antidepressants, antipsychotics, and diuretics. Prescribers should consider baseline cardiac vulnerability, exacerbation status, and cumulative QT burden.
- Clinicians should be cautious when prescribing theophylline due to its narrow therapeutic index and high potential for DDIs, making it less suitable for complex COPD treatment regimens in the elderly. By contrast, the newer roflumilast is not interaction-free but appears far less fragile than theophylline.
- Pharmacist involvement is essential for medication safety in COPD, particularly in complex regimens, because it addresses both pharmacologic and practical aspects of care. Community pharmacy screening may help identify regimens with substantial potential interaction burden before acute deterioration or hospitalization occurs [38]. Pharmacists can improve inhaler technique, adherence, and smoking-cessation support, while also contributing to early recognition of exacerbation-related problems and medication reconciliation across care transitions [95,96]. In hospitals, pharmacist interventions include drug review, inhaler assessment, patient education, and multidisciplinary coordination, with evidence suggesting benefit for symptoms, guideline-directed inhaler use, and rehospitalization risk [97,98,99]. Because electronic DDI checkers often disagree, pharmacist-led review should rely on clinical judgment complex cardiopulmonary regimens [100,101].
- The palliative and frailty literature illustrates that in advanced COPD, clinical implications include deprescribing, symptom prioritization, and goals of care. The literature on palliative care supports earlier incorporation of both symptom-focused and disease-specific approaches in the management of COPD due to worsening symptoms and treatment complexities [25,26,102,103,104]. In such patients, the best clinical intervention may be simplifying the regimen to align treatment with their functional goals and preferences.

**Figure 2:**
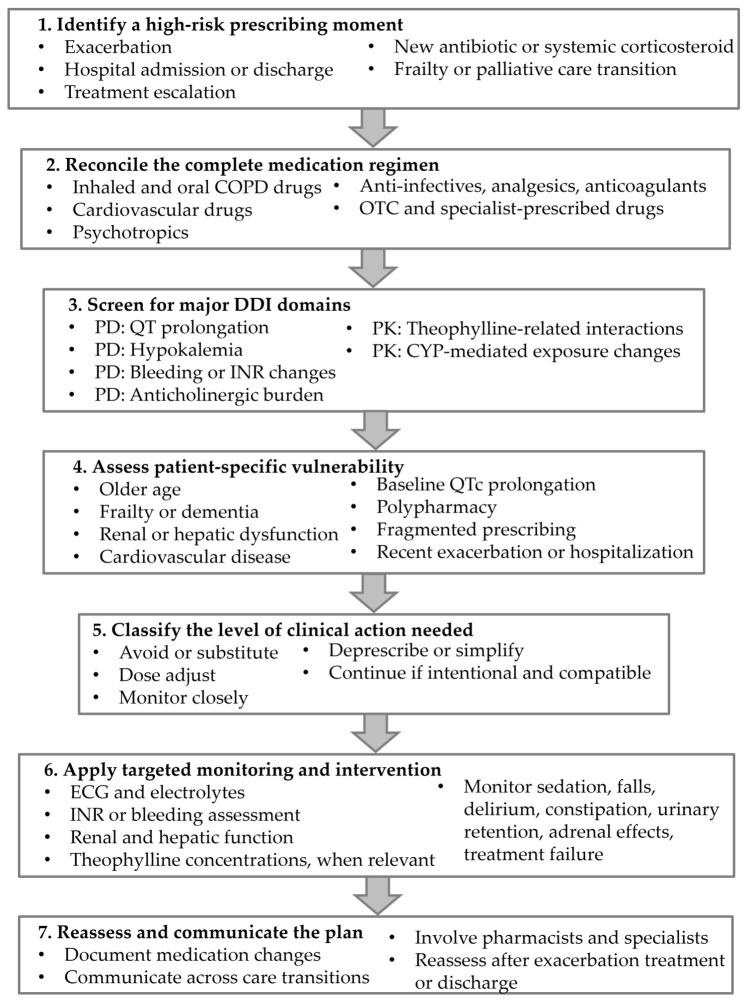
Practical pharmacist-supported algorithm for identifying and managing high-risk regimens in chronic obstructive pulmonary disease (COPD). The algorithm translates the regimen-level framework of this review into a clinically applicable sequence for structured medication review. It emphasizes recognition of high-risk prescribing moments, complete medication reconciliation, screening for major pharmacokinetic (PK) and pharmacodynamic (PD) DDI domains, assessment of patient-specific vulnerability, classification of clinical actionability, targeted monitoring and intervention, and communication across care transitions.

To improve clinical applicability, we translated the regimen-level framework proposed in this review into a practical pharmacist-supported algorithm for identifying and managing high-risk COPD regimens (Figure 2). This algorithm summarizes the key clinical steps that should follow recognition of a high-risk prescribing moment, including complete medication reconciliation, screening for major pharmacokinetic and pharmacodynamic DDI domains, assessment of patient-specific vulnerability, classification of clinical actionability, targeted monitoring and intervention, and communication across care transitions.

## 9. Limitations of the Evidence Base

The evidence regarding DDIs in COPD remains clinically useful but lacks methodological consistency. One significant limitation is that many studies assess potential DDIs and focus less on the clinically verified harm. This is especially true for checker-based cohort studies and hospital drug reviews, which are valuable for identifying recurrent interaction patterns but do not establish that every flagged combination caused toxicity, treatment failure, or adverse outcomes. In direct COPD cohorts, the proportion of severe or actionable combinations is lower than the total number of potential interactions generated by screening systems. This concern is reinforced by recent work showing only fair overall agreement between electronic drug interaction checkers, with agreement varying substantially across pharmacologic categories, indicating that DDI burden estimates from these checkers should be interpreted cautiously [100]. Furthermore, analyses of the evolution of drug-interaction databases indicate that DDI networks become denser over time and that standardizing severity remains a persistent challenge, which further complicates the comparative interpretation across different data sources [105]. Hence, research on DDIs associated with COPD may benefit from moving beyond pairwise analysis toward a regimen-level phenotyping approach capable of identifying clinically significant interaction-prone patient clusters in real-world multimorbid populations [106].

Another limitation is that many reported DDI signals in COPD overlap clinically with symptoms or outcomes caused by COPD progression, acute exacerbation, aging, frailty, or comorbid disease. Clinical findings such as dyspnea, tachycardia, arrhythmia, fatigue, falls, delirium, sedation, hospitalization, or mortality should therefore not be interpreted automatically as pharmacodynamic DDIs. Symptom-based associations without objective exposure, monitoring, or outcome data were interpreted as potential, not confirmed, DDIs [28,29,31]. Pharmacodynamic DDI signals were considered more robust only when supported by a plausible overlapping mechanism, temporal association with drug exposure, objective monitoring findings, or consistency across studies [28,29]. In the absence of such evidence, these signals were classified as potential pharmacodynamic DDIs, pharmacodynamic vulnerability, or regimen-level interaction burden.

A related limitation is the heterogeneity of definitions and methods across the COPD polypharmacy literature. Upadhyay et al. found that polypharmacy in COPD has been defined from at least 3 to at least 20 medications, with large variation in study settings, patient populations, and analytic approaches [14]. This matters because drug-count thresholds not only influence estimates of burden, but also how inhaled triple therapy, short-term exacerbation treatment, and multimorbidity treatment are counted and interpreted.

The reviewed evidence also varies substantially in design and clinical reliability. It includes case reports, retrospective reviews, observational cohorts, prescribing studies, product-label statements, healthy volunteer pharmacokinetic studies, and mechanistic or practice-focused reviews, with few controlled clinical studies conducted in representative COPD populations [28]. Large observational cohorts provide useful estimates of prescribing patterns and potential DDI burden but often cannot establish outcome attribution. Pharmacokinetic studies provide mechanistic exposure data but frequently involve healthy volunteers rather than older, frail, multimorbid, or exacerbating COPD patients. Case reports may identify clinically important signals but have limited generalizability. As consensus recommendations note, clinically relevant interaction evidence comes from diverse sources, which complicates direct comparison and requires careful interpretation of evidentiary strength [28].

Another limitation is that many clinically useful studies related to COPD are not strictly focused on DDIs, addressing multimorbidity, treatment burden, inappropriate prescribing, psychiatric management, palliative care, or respiratory-hospital pharmacotherapy. This fact highlights the complex relationship between DDI burden in COPD and non-respiratory prescribing, resulting in a scattered literature that often does not directly answer COPD-specific DDI questions.

Formal pharmacokinetic studies create a different kind of limitation. They typically involve healthy volunteers and do not address issues like frailty, polypharmacy, or disease severity seen in older COPD patients. Conversely, observational inpatient studies better reflect clinical populations, but often rely on screening tools and may lack validated outcome attribution.

In this context, physiologically based pharmacokinetic (PBPK) modeling can complement DDI prediction when clinical studies are infeasible, ethically challenging, or limited by conflicting evidence. PBPK models integrate drug-specific parameters, enzyme and transporter pathways, organ function, age, and disease-relevant physiology to estimate changes in drug exposure under defined co-medication scenarios. This approach is particularly relevant for CYP- or transporter-mediated DDIs involving theophylline, roflumilast, inhaled corticosteroids, and psychotropics. However, PBPK evidence remains mechanistic and predictive, not direct clinical outcome evidence. Its reliability depends on model qualification, verification against observed data, sensitivity analyses, and a clearly defined intended use. Therefore, PBPK modeling can complement clinical interpretation but cannot substitute for clinical assessment of pharmacodynamic risks, including QT prolongation, hypokalemia, sedation, bleeding, respiratory depression, or anticholinergic burden [55,107,108,109].

Finally, several clinically important subgroups remain undercharacterized. Frail older adults, nursing-home residents, patients with dementia, and those in advanced or palliative COPD are highly relevant to DDI risk. However, the available evidence for these groups is often limited to small studies, indirect data, or individual cases. Therefore, a narrative synthesis is appropriate, as there is enough evidence for meaningful interpretation but insufficient uniformity for a narrow quantitative model.

## 10. Conclusions

DDIs in COPD are best understood as regimen-level risks shaped by multimorbidity, therapeutic escalation, aging, and frailty, not only as isolated drug-pair events.

Future research on DDIs associated with COPD should report interactions in a more clinically interpretable and standardized manner. Studies should specify the exact drug combinations involved, clearly distinguish pharmacokinetic from pharmacodynamic mechanisms, indicate whether the interaction is expected to increase toxicity, reduce therapeutic efficacy, or both, and report its severity and practical actionability. More detailed information is needed regarding dose, route of administration, duration of co-exposure, treatment setting, and timing within the COPD trajectory, especially during exacerbations, hospitalization, and discharge. In addition, future studies should describe patient context more explicitly, including COPD severity, comorbidity burden, age, frailty, renal or hepatic dysfunction, and concomitant psychotropic or cardiovascular treatment, so that interaction findings can be interpreted in relation to real clinical vulnerability.

Studies of DDIs in COPD should also distinguish more carefully between harmful DDIs, intentional therapeutic combinations, and formal compatibility findings, particularly for inhaled regimens and multidrug maintenance therapy. More outcome-oriented research in representative COPD populations is needed to improve clinical decision-making and individualized risk reduction beyond simple prevalence estimates.

## Figures and Tables

**Table 1:** Qu antitative context from key studies relevant to drug–drug interactions (DDI) and regimen-level risk in chronic obstructive pulmonary disease (COPD).

Study	Population/Design	Quantitative Findings	Type of Evidence	Interpretation
Hanlon et al. [12]	UK Biobank cross-sectional study; COPD vs. no COPD	COPD participants had higher multimorbidity than non-COPD participants, 17% vs. 4%, and higher polypharmacy, 52% vs. 18%. COPD was associated with higher odds of receiving ≥3 drugs linked to falls, constipation, urinary retention, CNS depression, bleeding, and renal injury; ORs ranged from 2.22 for renal injury to 4.61 for bleeding.	Co-prescribing/ADR-risk burden	Supports regimen-level adverse-effect overlap; not a confirmed DDI outcome study.
Ierodiakonou et al. [32]	Primary-care COPD cohort, Crete; *n* = 245	Multimorbidity: 77.0%; polypharmacy: 55.2% overall and 62.4% in older adults; PIMs: 9.6% in those ≥65 years; cumulative co-medication risk: falls 22.3%, constipation 17%, cardiovascular events 12.8%; 15 major drug-to-drug interaction pairs in 11.5%.	Potential DDI and cumulative ADR-risk burden	Shows that interaction-related risk is detectable in community-managed COPD.
Graf et al./COSYCONET [40]	Large COPD cohort; *n* = 2741; baseline and follow-up	Median drug count ≥5 across patient categories; ≥3 non-respiratory drugs; PRISCUS-listed medication in 10.2% of patients ≥65 years; serious adverse drug combinations: 4.2%; potentially clinically relevant unwanted combinations: 6.4%; wanted combinations: 8.0%.	Potential DDI/compatibility classification	Useful because it separates serious, unwanted, and wanted combinations instead of treating all multidrug therapy as harmful.
Stojadinović et al. [41]	Hospitalized COPD patients; *n* = 71	Most serious pDDIs differed by checker: Medscape 22%, Epocrates 11%, Micromedex 49%. Each additional prescribed drug was associated with roughly doubled odds of serious pDDIs; number of prescribers OR: 6.44; Charlson Comorbidity Index OR: 7.78 in one model.	Potential DDI risk-factor study	Supports drug count, comorbidity burden, and fragmented prescribing as high-risk markers, but also shows checker-dependent variability.
Spanakis et al. [20]	Hospitalized respiratory patients; COPD exacerbation among common admission diagnoses; *n* = 102	Polypharmacy: 61% at admission, 98% during hospitalization, 63% at discharge. DDIs: 55%, 96%, and 63%, respectively. PD-DDIs were 81%; QT prolongation accounted for 31.4% of PD-DDIs; PK-DDIs were 19%.	Hospital pDDI burden; not COPD-exclusive	Supports hospitalization and discharge as high-risk medication-transition moments; should be labelled respiratory inpatient evidence.
Li et al. [42]	Hospitalized COPD patients in mainland China; *n* = 393	Polypharmacy: 96.9%; 640 DRPs identified; 56.7% had ≥1 DRP; treatment safety problems: 54.2%; antibiotics, corticosteroids, and PPIs were the top medication classes; 91.0% of pharmacist interventions were accepted and 91.6% of DRPs were solved.	Drug-related problems and pharmacist intervention outcomes	Supports the practical algorithm and pharmacist-led review; DRPs are broader than DDIs.
Elli et al. [43]	Italian nursing-home residents; COPD subgroup within 2604 residents	306 residents had COPD; 84 received ≥1 COPD medication; 45 treated residents had ≥1 potentially severe DDI, 53.6%; many involved QTc risk from beta-2 agonists with diuretics, antipsychotics, or antidepressants.	Potential severe DDI burden in frail/institutionalized patients	Supports frailty/dementia/nursing home vulnerability and the QT pharmacodynamic risk theme.
Zilberman-Itskovich et al. [44]	Hospitalized patients with AECOPD; *n* = 67	Prolonged QTc occurred in 35.8% at admission and 37.3% on day 3; hospital mortality was 12% in the prolonged QTc group and 0% in the normal QTc group.	Clinical risk marker; not necessarily DDI	Supports QTc as a COPD-relevant risk phenotype while avoiding over-attribution to DDIs.

Abbreviations: ADR, adverse drug reaction; AECOPD, acute exacerbation of COPD; CNS, central nervous system; COPD, chronic obstructive pulmonary disease; DDI, drug–drug interaction; DRP, drug-related problem; OR, odds ratio; PD, pharmacodynamic; pDDI, potential drug–drug interaction; PIM, potentially inappropriate medication; PK, pharmacokinetic; PRISCUS, German list of potentially inappropriate medications for older adults; QTc, corrected QT interval.

**Table 2:** Mechanistic framework for COPD-relevant drug–drug interaction assessment *.

Drug Class or Regimen Domain	Main Pathway or Mechanism	Interaction Mechanism	Potential Clinical Consequence	COPD-Relevant Context
Theophylline and other xanthines	CYP1A2 and CYP3A4 metabolism; narrow therapeutic index	Metabolic inhibition may increase exposure, while enzyme induction or smoking-related CYP1A2 effects may reduce exposure	Nausea, tremor, tachycardia, arrhythmia, seizures, toxicity, or loss of efficacy	Legacy therapy, older adults, polypharmacy, exacerbation-related anti-infective exposure
Roflumilast	CYP3A4 and CYP1A2 metabolism	Strong enzyme inducers may reduce roflumilast exposure; inhibitors may increase exposure	Reduced therapeutic efficacy or increased adverse effects	Severe COPD with chronic bronchitis and exacerbation risk; co-treatment with anti-infectives or enzyme-modifying drugs
Inhaled corticosteroids	CYP3A4 metabolism, especially for selected corticosteroids	Strong CYP3A4 inhibition may increase systemic corticosteroid exposure	Adrenal suppression, Cushingoid features, osteoporosis, infection risk	HIV therapy, azole antifungals, macrolides, multimorbidity, long-term inhaled corticosteroid exposure
Inhaled LABA/ beta-2 agonists	Predominantly local bronchodilator effect; systemic beta-adrenergic effects may occur, especially at high dose or during frequent rescue use	Additive sympathomimetic or potassium-lowering effects with diuretics, systemic corticosteroids, theophylline, stimulants, or duplicated bronchodilator therapy	Tachycardia, hypokalemia, QT-related vulnerability, arrhythmia risk	Exacerbation treatment, frequent rescue therapy, cardiovascular comorbidity, high-dose bronchodilator exposure
Inhaled LAMA/anticholinergics	Predominantly local bronchodilator effect; low systemic exposure	Additive pharmacodynamic anticholinergic burden with systemic anticholinergic or anticholinergic-like drugs	Dry mouth, constipation, urinary retention, delirium, glaucoma risk	Older adults, frailty, dementia, renal impairment, concomitant psychotropics or bladder antimuscarinics
Macrolides and fluoroquinolones	CYP3A4 inhibition for selected macrolides; pharmacodynamic QT liability	Metabolic inhibition of susceptible co-medications; additive QT prolongation	Increased exposure of co-medications, arrhythmia risk, treatment-limiting toxicity	Acute exacerbations, hospitalization, cardiovascular disease, psychotropic or antiarrhythmic co-medication
Azole antifungals	CYP3A4 inhibition; transporter effects for selected agents	Increased exposure of CYP3A4 or transporter substrates	Corticosteroid toxicity, psychotropic toxicity, QT risk, bleeding risk with susceptible anticoagulants	Pulmonary fungal disease, steroid-exposed lungs, structurally diseased lungs, complex multimorbid regimens
Psychotropics	CYP2D6, CYP3A4, CYP1A2; CNS and QT-related pharmacodynamic effects	CYP inhibition, poor metabolism, or interacting co-medications may increase psychotropic exposure; additive sedation, QT, or anticholinergic effects may occur	Sedation, falls, delirium, extrapyramidal effects, QT prolongation, respiratory symptom misinterpretation	Anxiety, depression, dementia, frailty, palliative care, hospitalization, exacerbation-related prescribing
Anticoagulants and antiplatelets	CYP3A4, CYP2C9, P-gp, BCRP, or other pathway involvement depending on agent	Metabolic or transporter inhibition/induction may alter exposure; additive bleeding risk may occur with antiplatelets, NSAIDs, corticosteroids, or interacting anti-infectives	Bleeding, INR instability, thrombotic risk if exposure is reduced	Cardiovascular comorbidity, atrial fibrillation, infection treatment, discharge medication changes
Statins and cardiovascular drugs	CYP3A4, OATP, P-gp, or other pathways depending on agent	CYP or transporter inhibition may increase exposure; pharmacodynamic overlap may affect heart rate, blood pressure, or conduction	Myopathy, rhabdomyolysis, bradycardia, hypotension, conduction abnormalities	Multimorbid COPD, cardiopulmonary co-treatment, calcium channel blockers, antiarrhythmics, polypharmacy
Benzodiazepines, sedatives, and opioids	CYP3A4 and CYP2D6 for selected agents; central nervous system depression	Increased exposure through metabolic inhibition; additive pharmacodynamic sedation or respiratory depression	Sedation, falls, delirium, hypoventilation, aspiration, respiratory depression	Advanced COPD, severe dyspnea, anxiety, palliative care, frailty, concurrent psychotropics
Diuretics and systemic corticosteroids	Pharmacodynamic electrolyte and metabolic effects	Additive potassium loss, fluid and metabolic effects, or interaction with beta-2 agonist exposure	Hypokalemia, QT-related vulnerability, arrhythmia risk, glucose or fluid-balance complications	Exacerbation treatment, heart failure, cardiovascular comorbidity, systemic steroid bursts

* Mechanistic domains and pathway assignments were summarized from regulatory DDI guidance and COPD/pharmacology studies [15,18,24,27,55]. Abbreviations: BCRP, breast cancer resistance protein; CNS, central nervous system; COPD, chronic obstructive pulmonary disease; CYP, cytochrome P450; DDI, drug–drug interaction; HIV, human immunodeficiency virus; INR, international normalized ratio; LABA, long-acting beta-2 agonist; LAMA, long-acting muscarinic antagonist; NSAID, non-steroidal anti-inflammatory drug; OATP, organic anion-transporting polypeptide; P-gp, P-glycoprotein; QT, electrocardiographic QT interval.

## Data Availability

No new data were created or analyzed in this study. Data sharing is not applicable to this article.

## Abbreviations

The following abbreviations are used in this manuscript:

ADR	Adverse drug reaction
AECOPD	Acute exacerbation of chronic obstructive pulmonary disease
BCRP	Breast cancer resistance protein
CAT	COPD Assessment Test
CNS	Central nervous system
COPD	Chronic obstructive pulmonary disease
CVD	Cardiovascular disease
CYP	Cytochrome P450
DDI	Drug–drug interaction
ICS	Inhaled corticosteroid
LABA	Long-acting beta2-agonist
LAMA	Long-acting muscarinic antagonist
MRCI	Medication Regimen Complexity Index
OATP	Organic anion-transporting polypeptide
PBPK modeling	Physiologically based pharmacokinetic modeling
PDE4	Phosphodiesterase 4
PK	Pharmacokinetic
QTc	Corrected QT interval
